# Enhancing hospital quality management and patient safety in Vietnam: a technical assistance project utilizing online solutions during COVID-19 pandemic

**DOI:** 10.1186/s41182-022-00435-2

**Published:** 2022-07-12

**Authors:** Jun Moriyama, Tomoo Ito, Masahiko Doi, Kaori Seino, Duong Huy Luong, Azusa Iwamoto, Hitoshi Murakami

**Affiliations:** 1 Bureau of International Health Cooperation, National Center for Global Health and Medicine, 1-21-1, Toyama, Shinjuku-ku, Tokyo, 162-8655 Japan; 2 Quality Management Division, Medical Service Administration, Ministry of Health, Room 711, 138A Giangvo, Hanoi, 11155 Vietnam

**Keywords:** COVID-19 pandemic, Hospitals, Online system, Technology, Smartphone, Vietnam, Quality management, Patient safety, Training

## Abstract

Since 2015, the National Center for Global Health and Medicine in Japan has been conducting a technical assistance project for improving patient safety in Vietnamese hospitals. During the COVID-19 pandemic, the project conducted a patient safety training program utilizing online solutions for participants from Vietnam. This resulted in an increase in the number of participants, and ensured access from remote locations. The convenience of easy access from smartphones encouraged further participation. In addition to online training, the utilization of platforms such as Facebook, a common social networking service in Vietnam, contributed to the dissemination of good practices.

## Introduction

Quality healthcare services are essential to achieving Universal Health Coverage included in Sustainable Development Goal (SDG) 3 (“Ensure healthy lives and promote well-being for all at all ages”) [1]. Aligned with SDG 3, World Health Organization is working toward ensuring patient safety which is the prevention of avoidable harm to patients caused by the process of healthcare [2]. However, it is estimated that one in 10 patients are harmed while receiving hospital care in high-income countries [3]. In low- and middle-income countries, the number of adverse events is still unclear [4].

In 2013, the Ministry of Health Vietnam issued Decision 19/2013 TT-BYT to implement hospital quality management under its leadership [5]. However, adhering to the Decision is not mandatory, the annual hospital audit conducted by the Ministry includes an evaluation item regarding establishment of a hospital quality management department, and if established and well managed, the hospital is highly evaluated. This system has encouraged Vietnamese hospitals to establish hospital quality management departments. The Decision also describes setting up a program and developing specific regulations to ensure a safe system such as identifying patients accurately, avoiding mistakes during provision of services, safe surgery procedures, safety in drug use, infection control and prevention, risk and error prevention due to miscommunication among healthcare workers, prevention of patient fall, and safe usage of medical equipment. Due to the increased awareness of patient safety in Vietnamese hospitals and the need for technical assistance, the National Center for Global Health and Medicine (NCGM), Japan, has been conducting a project to improve patient safety in Vietnamese hospitals. NCGM takes on various roles: conducting basic medical research directly related to hospitals and clinical practice, promoting international medical cooperation, and developing human resources. Authors of the current study are technical officers working at NCGM and the Ministry of Health, Vietnam.

The project, funded by the Japan’s Ministry of Health, Labour and Welfare, aims to share Japanese health care experiences and knowledge to improve public health and medical standards by transferring the organizational management and clinical skills on patient safety. The project objective is to improve the quality of healthcare service in Vietnamese hospitals through human resource development and the establishment of platforms to disseminate good practices.

The technical assistance provided can be divided into three parts: (1) organizing training in Japan and forums in Vietnam; (2) conducting online training during the coronavirus disease 2019 (COVID-19) pandemic; and (3) support for activating platform to share experiences in Vietnam.

## Approaches

### Organizing training in Japan and forums in Vietnam

Project participants include persons in charge of the quality management departments and staff working at the clinical departments of Vietnamese hospitals. Before the COVID-19 pandemic, the project had nominated seven to eight Vietnamese trainees annually and invited them to Japan for a 2-week training on patient safety since 2015 [6]. The project, in consultation with the Ministry of Health, Vietnam, nominated hospitals that are currently making advance in and those hospitals whose executives are highly interested in patient safety. Between 2015 and 2019, 44 trainees from 29 hospitals received face-to-face training in Japan. The 2-week training program in Japan included field visits, face-to-face lectures, and the development of an action plan according to the trainees’ hospital situation. The lectures included the medical safety management system in Japanese hospitals, leadership, incident reporting and learning system, multi-disciplinary team approach, and medical safety measures in related departments such as pharmacy, radiology, and clinical laboratory in NCGM. The training program was developed based on the Ministry of Health’s decision regarding the activities of the hospital quality management department, and what they would like to improve in their hospitals. The program was updated through discussions with the participants during the implementation of the training from 2015.

Additionally, to widely disseminate its experiences and good practices on patient safety, the project had been providing funds to hold patient safety forums in Vietnam. Before the COVID-19 pandemic, all five forums were held face-to-face in Vietnamese hospitals. The medical staff working in Vietnamese hospitals were invited to these forums, which were hosted by the Ministry of Health, public hospitals in Vietnam, and NCGM.

### Conducting online training during the COVID-19 pandemic

The COVID-19 pandemic made it difficult for trainees to travel between Japan and Vietnam. However, COVID-19 was controlled in Vietnam and Vietnamese people could travel within the country in 2020. In particular, Vinh Phuc General Hospital was highly regarded as the first hospital to control COVID-19 successfully. Therefore, the project organized a 5-day patient safety training in Vinh Phuc Province to learn not only from Japanese experiences, but also from these valuable Vietnamese experiences. To avoid COVID-19 infection risk, the project planned two ways of participation: gathering on-site at Vinh Phuc Province and participating online. Japanese lecturers joined the training online. Four of the five days were spent on online live lectures, and the remaining day was spent on the action plan presentation. The 4-day training was open-access for people interested in. For further understanding of the COVID-19 measures in Vinh Phuc General Hospital, participants who had gathered on-site visited the hospital and discussed their observations. After the training, action plans were developed by participants to improve their hospital situations. The project then evaluated these action plans with using the SMART (Specific, Measurable, Achievable, Related, Time-bound) criteria, which is often used for appropriate goal setting.

### Support for activating platform to share experiences in Vietnam

In Vietnam, academic conferences in the field of patient safety are limited, and there are few opportunities to share information among hospitals. Facebook is a common social networking service (SNS) in Vietnam [7], and the graduates who had participated in the training established a Facebook group to share their experiences with people concerned about hospital quality management and patient safety. NCGM provided information on patient safety training and the seminar.

## Outcomes and impacts

### Increasing the number of trainees through online training

**Fig. 1:**
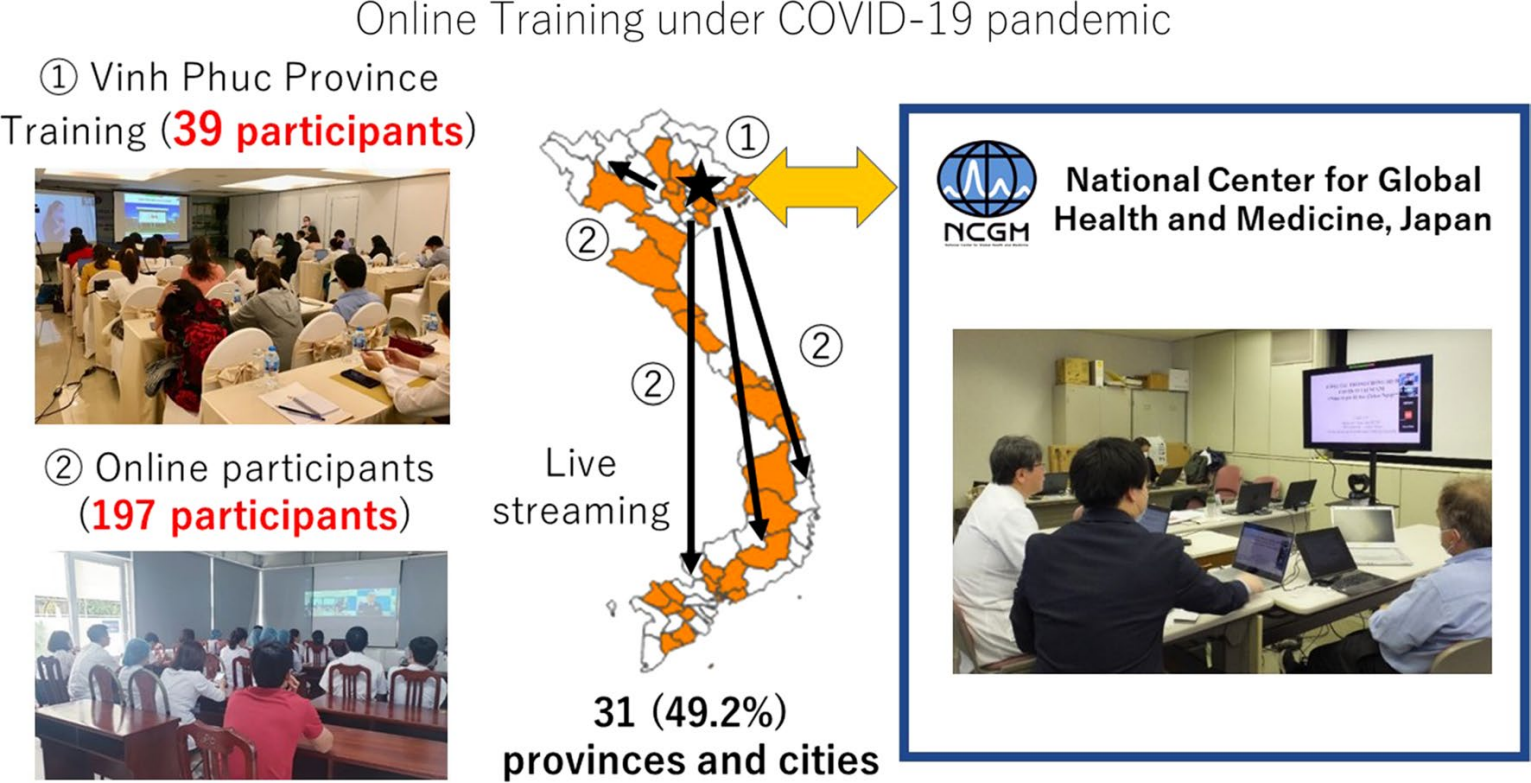
Conducting online training during the COVID-19 pandemic

Between 2015 and 2019, 44 trainees from 29 hospitals visited Japan. However, for the online training in 2020, 39 participants from 19 hospitals joined the training program in Vinh Phuc Province, and 197 participants joined from online devices such as smartphones or hospital computers. For those who participated online, their attendance time was checked using the online web conference system and they were subsequently issued attendance certificates. Table 1 shows the number of participants from 2015 to 2020. Figure 1 shows the overview of the training and the geographical distribution of the 197 participants who joined the training. Vietnam has 58 provinces and five centrally controlled cities. Online participants joined from 31 (49.2%) areas. The map was created by an open source, the quantum geographic information system 3.16.13 [8], to depict the geographical distribution of participation all over Vietnam.

**Table 1:** Number of participants from FY2015 to FY2020

Fiscal year (FY)	2015	2016	2017	2018	2019	2020
The number of trainees who participated in the training (location)	14* (Japan)	8 (Japan)	7 (Japan)	8 (Japan)	7 (Japan)	39 (Vinh Phuc Province, Vietnam) 197 (online)
The number of participants who joined in Vietnam Forum	58	98	223	344	286	–

*Two trainings were held in FY2015

### Results of the online training questionnaire

The project administered an online questionnaire to the 395 applicants who had registered for the training in 2020. The questionnaire aimed to assess the appropriateness of online training, including the program and online access. The questionnaire items included: achievement of the training objectives, devices used to participate, ease of use of the online conferencing tool, and timing of participating in the training. One hundred and seventy-two participants (43.5%) answered the questionnaire with a Likert scale. Regarding “achievement of training objectives”, 92 (53.5%) answered “Achieved”. For “devices used for the training”, 106 (61.6%) answered smartphone. For “ease of use of the training tool”, 100 (58.1%) answered “easy”. Concerning “timing of participating in the training”, 82 (47.7%) answered “in my free time while working in the hospital”.

### Forming of online platforms through SNS

The Facebook group focusing on patient safety that the trainees had established 23,852 followers (as of March 2, 2022). Additionally, the group was linked to the website of the Department of Medical Services Administration, Ministry of Health, Vietnam.

### Contributing to patient safety policies in Vietnam

Some trainees were nominated as government members for drafting a notification on the introduction of an incident reporting system in Vietnamese hospitals.

## Discussion

### Coverage of the project in Vietnamese hospitals

According to the Health Statistics Year Book 2018 issued by the Ministry of Health, Vietnam [9], there are 1409 hospitals in Vietnam, including both public and private hospitals. Although the project covered 98 hospitals (6.96%), it has been collaborating with the Ministry of Health, Vietnam, to select hospitals that could become leaders in promoting patient safety in Vietnam.

The project also focuses on supporting the introduction of safe systems according to the trainees’ hospital situation. For example, past trainees had developed an action plan to introduce or strengthen incident reporting and learning systems that collects medical incidents, analyzes their causes, and shares measures not to prevent similar incidents from occurring. Some trainees reported that the systems had been introduced successfully, and the number of reports had increased.

### Improving access from remote areas with the online system

Due to travel restrictions caused by the COVID-19 pandemic, the project switched to an online training program. The 197 people who participated in the training online came from 31 provinces and cities in Vietnam, out of 63 (58 provinces and five centrally controlled cities). While face-to-face training had to limit the number of attendees because of the capacity and cost of the venue, the number of participants increased in the online training, and access from remote locations was facilitated. As the questionnaire results indicated, the convenience of easy access from smartphones and easy to use online system may also have encouraged participation. It is also reported that e-learning has improved the quality and quantity of medical education, while reducing the burden on participants [10]. Online training has the potential to spread quality-assured education to a wider audience, thus bridging the gap between urban and rural areas.

### Providing opportunities to participate in the training for busy clinical staff

Learning about patient safety is essential for all healthcare staff. However, the staff working in the clinical departments are mostly busy to spare time for training. The online system however enables participation from their workplaces and homes. Furthermore, online training preempted the risk of COVID-19 infection from face-to-face contact.

### Promoting experience sharing by utilizing SNS

In addition to the online training, the utilization of technology such as SNS contributed to the dissemination of good practices. Facebook is a common SNS for sharing information and networking in Vietnam. The training graduates voluntarily created a Facebook group of 23,852 followers (as of March 2, 2022, indicating high interest in the topic of patient safety and quality improvement. Since there are only a few opportunities to share information in this field, it is necessary to facilitate information sharing through SNS.

### Limitations of online training

While the online systems are easy and convenient tools, there are limitations of online training. It has been reported that online administrators and students must have skills related to utilizing online tools and that stable internet connection is necessary and costly [11]. Evidence on online training for health care providers is limited and further validation of training effectiveness is required [12]. In this project, it was difficult to evaluate the effectiveness of online training when compared to face-to-face training in Japan.

### Possibilities of online training

Finally, with the project having adapted to online training due to the COVID-19 pandemic, training contents were disseminated to wide geographical areas. These efforts are important in achieving not only SDG 3, but also SDG 4, “Ensure inclusive and equitable quality education” [13]. In that regard, it is vital to continue developing effective ways of utilizing technology. However, while international cooperation in patient safety is in high demand, information is limited. Therefore, in addition to Japan, Vietnamese hospitals should promote sharing experiences with other countries to improving patient safety.

## Data Availability

Not applicable.

## Abbreviations

COVID-19: Coronavirus disease 2019
NCGM: National Center for Global Health and Medicine, Japan
SDGs: Sustainable development goals
SNS: Social networking service
